# Supplementary material for: Acceptance and compliance with micronutrient powder (MNP) among children aged 6–23 months in northern Nigeria
Source: PLOS Glob Public Health. 2022 Oct 17;2(10):e0000961. doi: 10.1371/journal.pgph.0000961 (PMC10022258; doi:10.1371/journal.pgph.0000961)
Supplement: S1 Dataset — (ZIP) [file pgph.0000961.s008.zip › S8_Dataset/Spot Checks.zip!/Spot Checks/110715_A_GU_PM_2_SC.pdf]

SPOT CHECK RECORDING FORM

State: __ADAMAWA___ LGA: __GUYUK_______ Community: _GOMBAIN________

Sex of index child: __MALE___ Age of index child (in months): __13 MONTHS_____

Spot Check Information

1. **Date of MNP distribution to household** (circle option below based on LGA name above)**:**
   - **Kebbi:** BirninKebbi&Jega LGA: **June 16**
   - **Kebbi:** Danko-wasagu: **June 17**
   - **Adamawa:**Song, Ganye: **June 18, JUNE 24**
   - **Adamawa:**Guyuk **June 19**
2. **Date of Spot Check Observation: ___11/07/2015________________**
3. **Number of sachets remaining**(Ask the caregiver to show you and count the specific number of sachets remaining)**:**

   48

4. **Number of sachets that should remainusing one MNP sachet per day** (Analyst only)**:**

   44

5. **Number of sachets remaining / number of sachets should be remaining**(Analyst only)**:**

__48____ / ___44____ = ___109______ %

Probes

Question 1. You have **XX** MNP sachets remaining. Explain how easy or difficult it was to use the MNP product on a daily basis for your child 6 – 23 months? (Circle one answer)

Very Difficult **Difficult** **Easy** **Very Easy**

Question 2. Describe any specific challenges that you faced that made the MNP difficult to use on a daily basis?

The child was seriously stooling when I started given him the MNP but it has stopped. Though the child likes the MNP but I still have to give him myself. But now he is doing fine and he drinks the MNP.

Question 3. Describe specific aspects of the MNP product that you or your child enjoyed that made it easier to use on a daily basis?

i. The boy likes the powder
ii. He looks more healthy

Question 4. What questions do you have about the MNP based on your experience using it?

none

Question 5. What specific recommendations do you have that we can do as a program to make the MNP easier to use on a daily basis?

If possible let the MNP taste like sugar, it will be nice so as to facilitate the easy consumption of the MNP.
